# Feasibility of a home-designed respiratory rehabilitation program for chronic obstructive pulmonary disease

**DOI:** 10.1017/S1463423623000324

**Published:** 2024-01-30

**Authors:** Nidhal Belloumi, Chaima Habouria, Imen Bachouch, Meriem Mersni, Fatma Chermiti, Soraya Fenniche

**Affiliations:** 1 Pulmonology Department Pavilion 4, Abderrahmen Mami Hospital, Ariana, Tunisia; 2 Faculty of Medicine of Tunis, University of Tunis El Manar, Ariana, Tunisia; 3 Occupational and Environmental Medicine Department, Charles Nicolle Hospital, Tunis, Tunisia

**Keywords:** BODE index, chronic obstructive lung disease, effort, respiratory rehabilitation

## Abstract

**Background:**

According to international guidelines, respiratory rehabilitation (RR) for patients with chronic obstructive pulmonary disease (COPD) is a cornerstone of standard non-pharmacological treatment.

**Aims:**

To evaluate feasibility of a home-designed RR program and analyze its medium-term impact on respiratory parameters and quality of life.

**Methods:**

This was a prospective study involving 74 COPD patients enrolled in January 2019 and put on inhaled bronchodilator treatment associated with RR at home following a written protocol, for 16 weeks. The comparative statistical analysis highlights the difference before and after RR in terms of clinical and functional respiratory parameters as well as in terms of quality of life (assessed on the short form 36 (SF-36) questionnaire). The comparison involves RR-adherent patients versus non-adherent patients.

**Results:**

Mean age was 66.7 ± 8.3 years with a median of 67 years. All patients were smokers, out of which 42 patients (57%) did not quit yet. Forty-one percent of patients were frequent exacerbators. The average COPD assessment test (CAT) score in our patients was 23. The average 6-minutes walk distance (MWD) was 304 m. The BODE index in our patients was 4.11 on average. The RR program was followed by 36 patients (48%). Thirty patients (40%) applied it at least twice a week. RR-adherent patients had an average CAT score decreasing from 23 to 14.5 (*P* = 0.011). Their average 6-MWD was 444.6 m by the end of the study, which would be 64.2% of the calculated theoretical value. The average FEV1 increase after RR was 283 mL. The majority (69%) of RR-adherent patients were ranked as quartile 1; BODE index ≤2. The average scores of physical, psycho-social, and general dimensions assessed on the SF-36 questionnaire improved in RR-adherent patients.

**Conclusions:**

RR is a key non-pharmacological treatment for COPD. Its interest originates from its multidisciplinary nature, hence its effectiveness in several respiratory parameters. Our study reflects the feasibility of home-designed protocols in the absence of contraindications. We highlight also the positive impact on quality of life after RR at home.

## Introduction

In 2010, the World Health Organization estimated that chronic obstructive pulmonary disease (COPD) affected nearly 348 million people worldwide (“Chronic Obstructive Pulmonary Disease (COPD),” n.d.). This prevalence is increasing due to the aging of the population. COPD is considered to be a general disease with a respiratory starting point and multi-systemic impacts, including peripheral and respiratory muscle dysfunction. A review of the disease severity classification was conducted by the Global Initiative for Obstructive Lung Diseases (GOLD) expert groups in 2016: COPD patients were classified according to the intensity of dyspnea, symptoms of the disease, and the risk of severe exacerbations (“Global Initiative for Chronic Obstructive Lung Disease – Global Initiative for Chronic Obstructive Lung Disease – GOLD,” 2019). Patients with COPD are generally sedentary compared to people of the same age group. They are held back because of a growing phobia of physical activity created by a phenomenon of “the spiral of dyspnea.” Physical abilities of patients are affected and physical activities are reduced in terms of harmony and maintenance, revealing a deficit in endurance, competition, and balance (Hernandes *et al.*, 2009; Donaire-Gonzalez *et al.*, 2013). Shrinkage of lower limb muscular fibers which contains fewer mitochondria has been reported to describe muscular deconditioning (Shrikrishna *et al.*, 2012; Natanek *et al.*, 2013). Performing respiratory rehabilitation (RR) including an exercise-based physical training program is considered a key pillar of COPD management (Spruit and Wouters, 2007; Evans and Steiner, 2017). The experts reported a wide benefit of lung rehabilitation on physical performance and on specific indicators of COPD in all stages regardless of any co-morbidities (Spruit, 2014; Houben-Wilke *et al.*, 2017). Despite the known benefits of RR, adherence to such programs is low. One of the main barriers to referral to RR for COPD is the lack of specialized centers especially in developing countries. Home-based RR was designed as an alternative to remove certain “barriers” that prevent patients from adhering to the programs of RR.

Simplifying, explaining, and supervising this therapy are the only guarantors of its integration into a daily life pattern and its long-term continuation. Home-designed RR was originally thought to maximize patient’s self-confidence, remove “psychological barriers,” and increase long-term adherence. The application of physical mild-intensity training at home without resorting to a dedicated center facilitates the assimilation of the principle of change in behavior toward a more favorable state to health. Since the content of rehabilitation programs is based on daily activities, the demonstration avoids the patient’s preconception reaction, which is itself the source of prejudice (K. Johnston *et al.*, 2013; K. N. Johnston *et al.*, 2013). The effectiveness of a home rehabilitation program is not demonstrated on a large population scale and in long term. The objective of our study was to analyze the feasibility and adhesion of COPD patients to a simplified home RR program and evaluate its impact in the medium term on the functional and quality of life of adherent patients.

## Methods

### Type of the study: study protocol

It was a prospective open label study conducted over 1 year, from January to December 2019, including COPD patients in Pulmonology Department Pavilion VI at Abderrahmen Mami Hospital, Tunisia.

### Patients

Population study included confirmed COPD patients after their consent. We made no restriction correlated with age, demographical parameters, or smoking habit. We adopted COPD definition published in late versions of the GOLD guidelines (“Global Initiative for Chronic Obstructive Lung Disease – Global Initiative for Chronic Obstructive Lung Disease – GOLD,” 2019). COPD is a common, preventable, and treatable disease that is characterized by persistent respiratory symptoms associated with a persistent airflow limitation that is due to airway and/or alveolar abnormalities usually caused by significant exposure to noxious particles or gases. The persistent limitation of the airflow is confirmed at spirometry by a Tiffeneau index <70% after inhalation of short-acting β agonists (SABA).

#### Inclusion criteria

- A spirometry showing a fixed obstructive disorder. The obstructive disorder is defined by a Tiffeneau index inferior to 70%. This entity is qualified as fixed if forced expiratory volume at the first second (FEV1) had shown less than 12% reversibility and less than 200 mL of increase after inhaled SABAs application.
- Patients put on inhaled long-acting bronchodilators
- Written informed consent to participate

#### Non-inclusion criteria

- Broncho-pulmonary sequellae which generates an obstructive respiratory disorder
- Severe chronic respiratory failure which necessitates a home oxygenotherapy treatment
- Respiratory exacerbation during inclusion period
- Instable chronic disease which affects quality of life or counter-indicates the practice of a 6-minute walk test (6-MWT)
- Recent anginous thoracic pain or intermittent vascular claudication

#### Exclusion criteria

- Instable hypertension, electrocardiographic abnormalities, or oxygen desaturation <90% discovered prior to 6-MWT
- Recent anginous thoracic pain or intermittent vascular claudication occurring during 6-MWT
- Withdrawal of consent
- Contraindications of the functional respiratory tests

### Study protocol

Inclusion period lasted from January to April 1st, 2019. Our study included, from February to April 2019, all consenting COPD patients, who have been followed and treated by our medical team and who met the inclusion criteria. Assessment of the respiratory function was done through a clinical evaluation of symptoms, using mMRC scale of dyspnea, COPD assessment test (CAT) score for COPD symptoms, and then a spirometry, a 6-MWT, and a calculation of BODE index at the beginning of the study. BODE index included body mass index, decline of FEV1, severity of dyspnea, and performance during exercise. Quality of life was assessed by the Arabic Tunisian version of short form 36 (SF-36) (Ware and Sherbourne, 1992; Guermazi *et al.*, 2012). Data gathered included smoking habit, past medical history, COPD severity and stage of the disease, exacerbation’s frequency in 2018, inhaled treatment, and adherence to treatment (Morisky questionnaire). Following this first assessment, the home-designed rehabilitation protocol was explained for patients: stretching exercises, warm-up, endurance and limb strengthening exercises, water drink, and hyper protein diet for lean patients. The explanations were oral and written in a four-page manual. This manual also contained a calendar on which our patients were reporting the dates when they followed the program. No randomization was done; all patients received the same instructions and were asked to perform the rehabilitation program.

After 4 months, a second appointment was scheduled for every patient. After verification of the exclusion criteria, we checked for adherence to the home-based rehabilitation program: exercises practiced, exercises avoided, and rhythm of practice of the exercises. Subsequently, patients had a spirometry assessment, a 6-MWT, and a second SF-36 quality of life assessment.

In 6-MWT, instructions are standardized and should be clear and concise (ATS Committee on Proficiency Standards for Clinical Pulmonary Function Laboratories, 2002). The formulae used to calculate the predicted distance were validated and published by Ben Saad e*t al.* (2009). The walked distance (D) is mentioned by meters or as a percentage compared to PD.

Predicted walked distance (PD) by meters = [−160.27 × sex (0: man; 1: woman)] − [5.14 × age (years)] − [2.23 × weight (kg)] + [2.72 × height (cm)] + 720.50.

Quality of life assessment was performed using the SF-36 questionnaire. It contains 8 dimensions and 36 questions or items. Dimensions are physical functioning (PF), role physical (RP), bodily pain (BP), general health (GH), vitality (VT), social functioning (SF), role emotional (RE), and mental health (MH). One item, the question number 2 discusses the progress of the general health. Answers were converted to scores from 0 to 5, and then dimensions get scores from 0 to 100.

### Home-designed protocol of RR

Within the protocol, we insisted about stretching exercises, warm-up followed by an endurance exercise (shuttle walk or running), limb strengthening, and abdominal wall re-building exercises. Other components included rehydration, hyperprotidic diet, encouragement for smoking cessation, and adherence to treatment. Self-management and self-monitoring were respected. Patients were asked to perform two sessions per week at least and fill a diary with the corresponding dates when the rehabilitation was done.

### Ethical approval/statistical/statistical study

The main objective was to compare the respiratory functional parameters among rehabilitation adherent patients (RA) versus non-adherent (RNA) patients, so that we could assess the real benefit of this protocol of non-pharmacological treatment. The secondary objective was to assess the compliance of a representative group of COPD patients to a simplified home-designed RR protocol. The study protocol was discussed for approval with the local ethics committee in Abderrahmen Mami Hospital. The study was then submitted to the Pan African Clinical Trial Registry (PACTR) for approval. Study protocol number is PACTR202005764510617.

## Results

**Figure 1.**
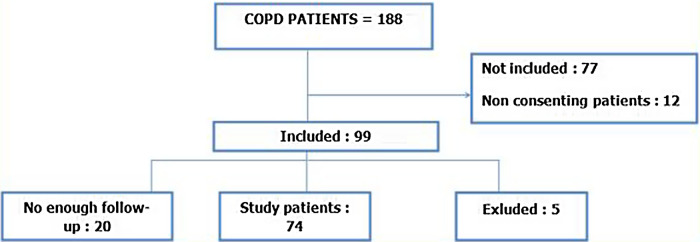
Flow diagram of the study process.

A total of 99 patients was included. After exclusion of five patients (for untreated hypertension, obliterate arteritis of the lower limbs, or atrial fibrillation) and loss of contact with 20 others, we kept 74 patients in the study (Fig. 1). The mean age was 66.7 ± 8.3 years.

All patients were active smokers. During inclusion, 42 patients (57%) were current smokers, and 32 patients (43%) had already quit smoking. Mean duration of the smoking habit was 43 years. Main comorbidities registered were hypertension (24%), diabetes mellitus (14%), gastric ulcer (14%), and dyslipidemia (9%).

### First assessment before rehabilitation

Dyspnea on exertion was evaluated using mMRC scale: 22% of patients had mild dyspnea, 36% of all patients had moderate dyspnea, 31% of all patients described it as severe, and 9% of patients had very severe dyspnea.

Mean CAT score was equal to 23, scores were ranging from 4 to 40. Ten patients (13%) were mild symptomatic patients (score CAT < 10).

In 2018, acute exacerbations of COPD (AECOPD) occurred zero to four times per patient. Forty-one per cent of all patients were frequent exacerbators (two exacerbations or more per year; Table 1). Severe exacerbations of COPD occurred zero to three times per patient. Thirty-one per cent of all patients had an admission for AECOPD in 2018.

**Table 1.** Patients distribution according to the frequency of AECOPD in 2018

Times per year	Exacerbation		Severe exacerbation	
	Number	%	Number	%
0	23	31	51	69
1	21	28	17	23
2	16	22	4	5
3	12	16	2	3
4	2	3	0	0

Considering the severity of dyspnea, CAT score, and exacerbation risk of occurrence, patients were classified as follows in Table 2.

**Table 2.** Spirometric findings, mobilizable respiratory volumes, and flux in COPD patients

Parameter	**Mean** ± **SD**	% of predicted value
**FVC (ml)**	2652 ± 691 [1080–4600]	74.2 [71–114]
**FEV1 (ml)**	1367 ± 482 [510–2880]	48.4 [21–91]
**FEV1/FVC (%)**	51.6	

FEV1 = Forced expiratory volume in the first second; FEV = Forced vital capacity.

Spirometric findings in 2018 revealed the following: mean FEV1 was 1367 ± 482 mL. Mean forced vital capacity (FVC) was 2652 ± 691 mL (Table 2).

**Figure 2.**
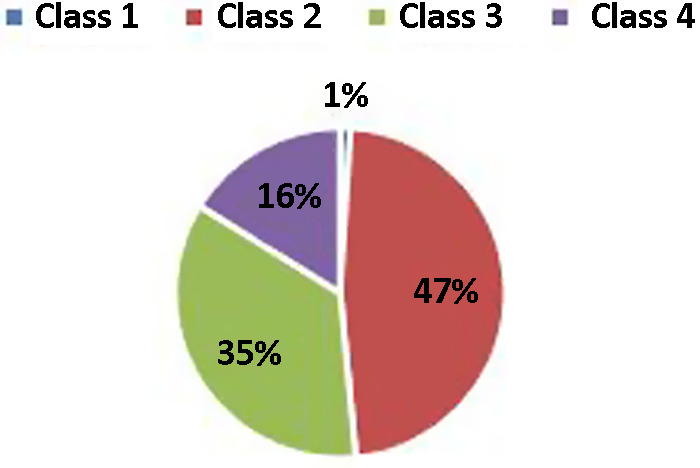
Spirometric classification of the study patients.

Spirometric classification of COPD, depending on FEV1 percentage of predicted value, had shown a majority of Class 2 patients (Fig. 2).

The mean distance in 6-MWT was 304 ± 126 m (110–610). The mean percentage of predicted values was 46%. Percutaneous oxygen saturation was 96.5% on average. A desaturation during exercise was registered in 37 patients (50%). Their mean distance in 6-MWT was 234.5 m, which corresponded to 35.6% of the predicted value. Patients who stopped during 6-MWT (21 patients; 38%) had a mean distance equal to 193.5 m, which corresponded to 29.5 % of the predicted value.

BODE index in our cohort was 4.11 in average. Twenty per cent of patients had an index of seven or over.

Inhaled long-acting β2 agonists (LABA) were prescribed for 65 patients (88% of all patients; Table 3). Long-acting muscarinic antagonists were prescribed for 25 patients (34%). Theophylline was always associated with LABA. Corticosteroid inhalers were prescribed for 16 patients (22%), always associated with inhaled bronchodilators. Adherence to treatment was noted in 54 patients (73%).

**Table 3.** Distribution of patients according to GOLD classification, BODE quartiles, and inhaled bronchodilator treatment

GOLD class	Number	%
GOLD A	9	12
GOLD B	7	10
GOLD C	3	4
GOLD D	55	74
BODE index/Quartiles	**Number**	**%**
0–2	21	28
3–4	24	32
5–6	14	19
7–10	15	20
Inhaled treatment	**Number**	**%**
LABA	16	22
LABA + LAMA	13	18
LABA + Theophylline	20	28
LABA + Theophylline + CSI	7	10
LABA + CSI	6	8
LAMA	9	12
LABA + LAMA + CSI	3	4

CSI = inhaled corticosteroids; LABA = long-acting β2 agonists; LAMA = long-acting muscarinic antagonists.

Quality of life decline in COPD patients was major in specific dimensions: “Role limitations due to physical health” whose score was 27.7 as average and “Role limitations due to emotional problems” whose score was 22.9 as average (Table 4). General health score was 79 maximum and 39.4 average.

**Table 4.** Mean scores of all SF-36 dimensions

Dimension	Mean	Median	Maximum	Minimum
Physical functioning (PF)	47	50	95	5
Role limitations due to physical health (RP)	**27.7**	25	100	0
Energy/Fatigue (VT)	49	50	85	5
Pain (BP)	66	65	100	10
**Mean of physical dimensions**	47.5	46.2	91.2	16.2
Role limitations due to emotional problems (RE)	**22.9**	0	100	0
Emotional well-being (MH)	76.3	80	100	28
Social functioning (SF)	56.9	62.5	100	0
**Mean of psychological dimensions**	52	52.6	100	17.5
**General health**	39.4	41.7	79	0

### Adherence to the RR program

We found 36 adherent patients (48% of all patients), who applied the rehabilitation program. Thirty patients (40%) stick to the program at least twice a week. Seven patients did it everyday. Jogging (or biking) was performed by all adherent patients. Arms strengthening exercises and anterior abdominal wall building exercises were done regularly by 17 patients (23%) and 8 patients (11%), respectively.

Mean age in RA group was lower than RNA group. Frequent COPD exacerbating phenotype was more frequent among RNA (Table 5; *P* = 0.021).

**Table 5.** COPD features in RA and RNA groups

Parameter		**RA** **(** ***n*** = **36)**	**RNA** **(** ***n*** = **38)**	***P***
Mean age (years)		63	**70**	**0.05**
mMRC	Stage 1 (%)	25	18	0.06
	Stage 2 (%)	48	27	
	Stage 3 (%)	25	37	
	Stage 4 (%)	0	18	
CAT score		23	24	0.16
Exacerbations per year	0 (%)	39	23	**0.021**
	1 (%)	33	23	
	≥ 2 (%)	28	**54**	
Stade GOLD	A	12	13	0.29
	B	11	9	
	C	4	3	
	D	70	77	
BODE index		3.8	4.4	0.11

mMRC = modified medical research council; CAT = COPD assessment test.

Among 42 active smokers, 19 patients quit smoking during the study progress. Seventeen patients among them followed the RR program, while the two others were in the RNA group. Patients still smoking till the study closure (23 patients) were predominantly in the RNA group.

### Impact of the home-designed RR on COPD patients

Mean mMRC score was 2.3 at the beginning and became 1.9 at the end of the study. Adherent patients had a dyspnea score passing from 2.4 to 1.55 on average. On the other hand, non-adherent patients had a dyspnea score passing from 2.1 to 2.5 on average.

Mean CAT score was 20.2 at the beginning and became 23 at the end of the study. CAT score in RA group dropped from 23 to 14.5 on average, the difference was statistically significant (Table 6; *P* = 0.011). CAT score in RNA group stand still in this study (24 before rehabilitation and then 25.6 at the end of the study).

**Table 6.** Mean mMRC score and CAT score after pulmonary rehabilitation in COPD patients

mMRC score	All patients		RA		RNA		P
	N	%	N	%	N	%	
0	6	8	5	7	1	1	
1	24	33	**19**	**26**	5	7	
2	20	27	10	13	10	13	<0.001
3	18	24	2	3	**16**	**22**	
4	6	8	0	0	6	8	
Total	74	100	36	49	38	51	

	All patients		RA		RNA		p
**CAT score**	N	%	N	%	N	%	
CAT < 10	9	12	**8**	**11**	1	1	
CAT ≥ 10	65	88	28	38	37	50	0.013
Total	74	100	36	49	38	51	

mMRC = modified medical research council; CAT = COPD assessment test.

**Figure 3.**
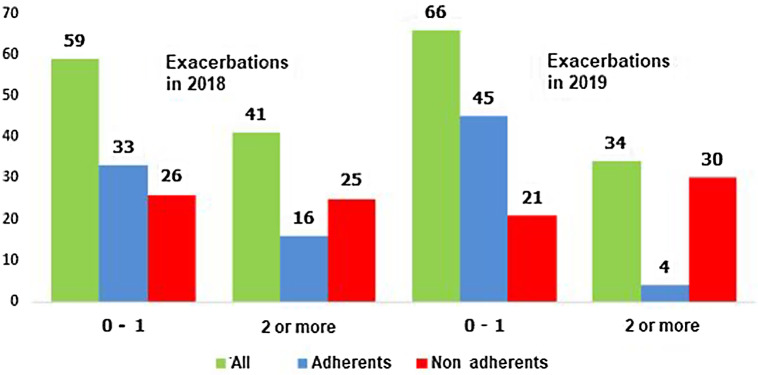
Distribution of patients according to the COPD exacerbations frequency in 2018 and 2019.

Frequent exacerbator phenotype was found in 41% and 34% of patients, respectively, in 2018 and 2019. This drop was mostly found among RA patients; exacerbators were 16% and 4% of patients, respectively, in 2018 and 2019 after the rehabilitation (Fig. 3; *P* = 0.021).

Mean FEV1 and FVC values were, respectively, 1510 ± 503 mL and 2750 ± 714 mL. Adherent patients had a mean FEV1 value equal to 1737 ± 470 mL. Non-adherent patient’s mean FEV1 value was 1300 ± 435 (*P* < 0.0001). Table 7 shows the difference in spirometric volumes (ΔFEV1 and ΔFCV) from the start to the end of the study.

**Table 7.** Outcome of the spirometric volumes with or without respiratory rehabilitation

Ventilatory parameter	All patients	Adherent patients	Non adherent patients	P
Mean ΔFEV1 (ml)	143	283	10	<10^−3^
Mean ΔFVC (ml)	97	222	−20	<10^−3^
Mean FEV1 (ml)	1510 ± 503	1737 ± 470	1300 ± 435	<10^−3^
Mean FVC (ml)	2750 ± 714	3020 ± 730	2494 ± 600	0.001

FEV1 = Forced expiratory volume in the first second; FEV = Forced vital capacity.

6-MWT mean distance was 356.2 ± 18.8 m (from 109 to 670 m), reaching 53.6% of the predicted distance. An oxygen desaturation during the test occurred in 23 patients (31%). Their mean distance was 227.4 m and represented 35.8% of the predicted distance.

RA patient’s mean distance in the 6-MWT was 444.6 m and represented 64.2% of the predicted calculated distance. RNA patient’s mean distance was 272.4 m corresponding to 43.5% of their predicted distance. The difference was significantly great (*P* < 0.0001). Desaturation during walking occurred in four patients among RA group versus 19 patients among RNA group.

**Figure 4.**
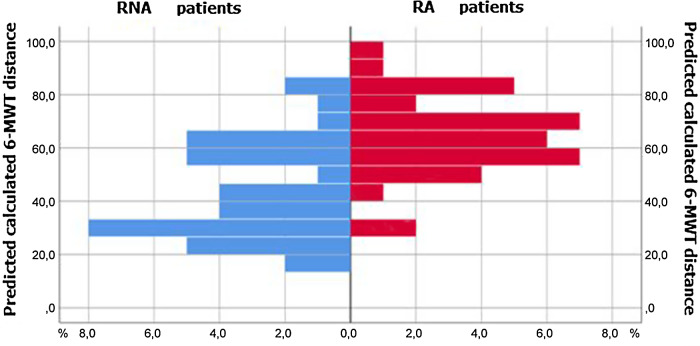
Patient’s distribution according to the 6-MWT distance (evaluation made after 16 weeks of rehabilitation).

After 16 weeks of rehabilitation, as shown in Fig. 4, RA patients improved their 6 minutes walking distance by 122.2 m (getting from 322.4 to 444.6 m).

BODE index of our patients was 3.2 on average; improvement after rehabilitation was obvious. Nine patients (12%) had an index of seven or above.

RA patient’s mean BODE index was 2, while NRA patient’s had a BODE index of 4.6 on average. The difference was statistically significant (Table 8; *P* = 0.008).

**Table 8.** Distribution des patients according to BODE index (evaluation made after 16 weeks of rehabilitation)

BODE index/Quartiles	**RA patients (** ***n*** = **36)**	**RNA patients (** ***n*** = **38)**
0 – 2	69	24
3 – 4	25	29
5 – 6	6	24
7 – 10	0	23

The impact of the pulmonary rehabilitation program on quality of life was obvious at the end of the study (Table 9). In SF-36 questionnaire compilation, mean scores of physical performance dimensions were enhanced, especially the dimension “Role limitations due to physical health” (RP), for which mean score passed from 27.7 to 42.6. Mean score of all physical dimensions passed from 47.5 to 57.1. RA patients had a significantly higher mean score of all physical dimensions, compared to RNA patients (Table 9).

**Table 9.** Mean scores of physical dimensions after 16 weeks of pulmonary rehabilitation program

Dimension – mean score	**All patients (** ***n*** = **74)**	**RNA patients (** ***n*** = **38)**	**RA patients (** ***n*** = **36)**
Physical functioning (PF)	57	**40.9**	**79.9**
Role limitations due to physical health (RP)	42.6	**18.4**	**68.1**
Energy/Fatigue (VT)	54.4	40.3	69.3
Pain (BP)	74.5	68.4	80.9
**Mean of physical dimensions scores**	57.1	**42**	**73**
Role limitations due to emotional problems (RE)	**41.9**	**20**	**64.8**
Emotional well-being (MH)	78.2	69.1	87.8
Social functioning (SF)	57.1	48	66.7
**Mean of psychological dimensions scores**	59	54.8	73
**General health**	46.8	41.7	52.2
**Outcome over one year**	48.3	**30.9**	**66.6**

Mean scores of psychological dimensions were enhanced, especially the dimension “Role limitations due to emotional problems” (RE), for which mean score passed from 23 to 41.9, reflecting a lesser eviction reflex and fewer unfinished patient’s wishes due to dyspnea worsening. Mean score of all psychological dimensions rised slightly from 52 to 59. RA patients had a higher mean score of all psychological dimensions, compared to RNA patients (73 versus 54.8).

Mean general health score did not vary significantly at the end of the study, considering all patients. Mean score was slightly better for RA patients than RNA patients.

However, 28 patients (37.8%) considered themselves worse globally than they were last year. This outcome was mentioned predominantly (21 cases) by patients who did not try the RR.

## Discussion

Our objective was to analyze the feasibility and adhesion of COPD patients to a simplified home RR program and to evaluate its impact after 16 weeks of practice on various parameters of the disease.

Our first evaluation immediately after the inclusion had showed a high frequency of dyspnea on exertion, described as moderate to severe. Also, CAT mean score was considerably high. Clinical assessment of COPD revealed a predominance of group D phenotype according to GOLD classification (“Global Initiative for Chronic Obstructive Lung Disease – GOLD,” 2019). Respiratory functional limitation was perceptible through the 6-MWT, showing a mean walked distance equal to 46% of the predicted one and a desaturation in 50% of all patients.

The analysis of the SF-36 questionnaire responses reflected the impact of the respiratory disease on physical abilities perceived by patients, psychological balance (self-confidence), and general health status perception.

The rehabilitation program was applied by 36 patients (48% of all patients). The adherence percentage would be better if we manage to make more encouragement to every patient by phone calls, mailing lists, or putting them into groups.

At the end of the study, patients who applied the program of rehabilitation perceived less dyspnea on exertion, milder COPD symptoms, higher spirometric mobilizable measured volumes, and better capacity of physical exercise. Mean scores of all dimensions of quality of life increased comparatively to the beginning.

Comparatively to RNA patients, dyspnea severity was lesser, 6-MW distance was better on average, and all components of quality of life were at a better level among RA patients.

Clinical characteristics of COPD patients in our study are comparable to previous prospective studies on pulmonary rehabilitation.

A substantial number of studies focused on the importance of the non-medicinal treatment for COPD patients, especially pulmonary rehabilitation. Most often, physicians describe protocols of physical exercise in dedicated centers with a monitoring and close follow-up (Cheng *et al.*, 2014).

International recommendations on chronic respiratory diseases endorse the role of RR for all patients without any restrictions related to the severity of the decline in respiratory function (Spruit *et al.*, 2013; Spruit, 2014; Garvey *et al.*, 2016, 2013). With the ultimate goal of changing behavior to a more healthy state, the rehabilitation program theoretically includes several components modulated by the caregiver to fit to the capabilities of the COPD patient. Seeking for a long-term adherence to this concept, physicians are aware of the need to encourage the patient and rise his confidence to change his lifestyle. Adherence would optimize and maintain the benefits acquired by RR protocol. The “new” goal is to promote autonomy of the patient, his capacity of exercise, and minimizing the restrictions generated by his respiratory disease (Ashworth *et al.*, 2005; Hageman *et al.*, 2018; Grosbois *et al.*, 2019; Nolan *et al.*, 2019; Simonÿ *et al.*, 2019; Hansen *et al.*, 2020).

In our study, ease of program delivery was ensured by maintaining flexibility in the choice of exercise intensity, repetition rate, and number of sessions per week to the patient himself. The rehabilitation program is first explained, negotiated with the patient who is responsible for adapting it according to his or her subjective respiratory abilities, after optimization of medical treatment. The program contains a first session of stretching and warming. Experts recommend checking balance and fall risk for patients with chronic respiratory failure (Spruit, 2014; Cox *et al.*, 2018).

The ideal number of repetitions is 8–12 repetitions for a given exercise followed by a break and then a restart. To promote aerobic metabolism during exercise, our patients were advised to increase the number of series without greatly increasing the number of repetitions and keep pauses between series. Our protocol includes mild intensity rectangular-type sessions of exercise (warming, constant intensity during effort, pause, and resumption) to guarantee a safer way to apply it at home with no medical monitoring.

Several studies already proved a better results after following a rehabilitation program with a personalized coaching, modulating in intensity of exercise, starting from mild to high rate practice, especially if the patient was included in a group of patients (Casaburi and ZuWallack, 2009; Leite *et al.*, 2018; Ciavaglia *et al.*, 2014). Building a team enables all members within it to move forward quicker toward high rate exercises with no hesitation. Authors suggest to start the pulmonary rehabilitation for all respiratory failure patients in a dedicated center until getting a sufficient immersion, an autonomy to go on at home with a maintained rate (Recommandations de la société de pneumologie de langue francaise, 2010; Spruit *et al.*, 2013; Spruit, 2014). Adopting home-based rehabilitation programs allow physicians to prescribe it to a huge number of patients, greater than what they could reach in rehabilitation center. Mild intensity programs with flexibility in changing the exercises intensity aimed at preserving patient’s safety, enhancing self-confidence, and getting to a maintained aerobic workout with progressively higher intensity exercises.

Multidisciplinary management during RR is more likely to be applied in dedicated centers. Those areas include kinesiotherapy for limbs or chest, dietetic management, tobacco cessation, psychological support when needed, and education. In our study, tobacco cessation was truly verified, therapeutic education was repeated in all appointments, and a hyper-protein diet and a sufficient hydration were reminded. Lahham *et al.* (2018) reported a fear to start a physical exercise and an avoidance reflex disabling COPD patients from getting used to pulmonary rehabilitation. In our study, we tried to bypass it by performing a 6-MWT at the beginning of the study. It was a moment of confrontation and demonstration to COPD patients. Once they try jogging, running, or cycling in a circuit of specified length in the neighborhood, self-confidence rise to permit a better adhesion to the RR program.

The patient was the unique guarantor of compliance. This simplified rehabilitation protocol was applied at home by 48% of COPD patients. The group of adherent patients was heterogeneous. It contained elderly, dyspneic or symptomatic people, and frequent exacerbating patients. Indeed, the severity of the disease was not a predictive parameter of adhesion of patients to rehabilitation at home nor of the subsequent response after 4 months of mild physical exercise. As long as a rehabilitation “profile” cannot be defined, it would be better to offer it to all patients with COPD, apart from an exacerbation, as long as there is neither safety constraint nor physical limitation.

Our study highlighted the beneficial effect of a RR program after 4 months, by analyzing two comparable groups of COPD patients, among them one group joined this program.

A striking result at the end of the study was an improvement of dyspnea at exercise among patients applying rehabilitation. This symptom is considered to drive the COPD patients to a deconditioning status. Dyspnea is directly linked to bronchial obstruction associated with pulmonary emphysema that hinders hematosis. Dyspnea becomes worse in severe COPD patients characterized by sarcopenia, osteoporosis, and depression. COPD patients who adhered to rehabilitation in our study had a mild dyspnea (26% of patients) at the end of the study. Exercise training is the method of choice to minimize dyspnea because it can neutralize its psychological and physiological components. Tolerance and adaptation to exercise at the muscular and cellular level is the most likely explanation of the mechanism by which training improves dyspnea, given that there is no significant change in cardiac or respiratory function in a few weeks of rehabilitation in most studies. The above-described adaptation is done on the mitochondria of the striated skeletal muscles including the main respiratory or accessory muscles. The increase in oxygen consumption (VO2Max) after re-training argues in favor (Cheng *et al.*, 2014). In our study, the 6-minute walking test data reflect improved fitness for effort and exercise. The average gain in walking distance in 6 minutes among patients enrolled in the RR program was 122.2 m, without desaturation or stopping the test. This result is as significant as the improvement of VO2Max because the 6-MWT is a typical endurance test and involves a sub-maximum force level. Daily activities require the same muscular performance. Training can also lead to better neuromuscular coordination, sought for our COPD patients because of its direct involvement in the patient’s autonomy.

The particularity of our study is the improvement of FEV1 in COPD patients after 16 weeks of rehabilitation at home (Table 10) versus a similar volume in the second group receiving bronchodilators with no rehabilitation. Nevertheless, most studies showed stable mobilizable volumes in cohorts of COPD patients who applied RR, even in medical centers or at home (Lan *et al.*, 2013; Cheng *et al.*, 2014; McCarthy *et al.*, 2015).

**Table 10.** Outcome of various ventilatory parameters after pulmonary rehabilitation in COPD patients

Ventilatory parameters	**Lan *et al.* (2013)**	**Cheng *et al.* (2014)**	Our study
ΔFEV1 (ml)	40	−30	**283**
ΔFVC (ml)	−30	10	**222**
ΔTiffeneau index (%)	2.1	−1.3	1.5
PiMax (cmH2O)	**7.8**	**11.4**	–
PeMax (cmH2O)	**12**	**12.4**	–
Duration of the rehabilitation program (weeks)	12	12	16

FEV1 = Forced expiratory volume in the first second; FEV = Forced vital capacity.

Some studies evaluated quality of life after RR and used dedicated scores for chronic respiratory diseases such as the Saint Georges Respiratory Questionnaire or the Clinical COPD Questionnaire. The authors noted an improvement in total scores within 6 weeks (Griffiths *et al.*, 2000; Lan *et al.*, 2013). The beneficial effect of RR on quality of life was obvious even 1 year after starting it. All dimensions of quality of life (total score, activity score, symptom score, and dyspnea impact score) were improved in the COPD cohorts after rehabilitation (Güell *et al.*, 2000; Lan *et al.*, 2013). Our study finds similar results using the SF-36 score (Table 11).

**Table 11.** Quality of life enhancement in COPD patients after pulmonary rehabilitation

Study	Training protocol	Number/Duration	Quality of life assessing score/Results
Lan *et al.* (2013)	Rehabilitation center	26 patients/12 weeks	Score SGRQ/enhancement of the scores of physical activity, fewer symptoms, better dyspnea perception
Lake *et al.* (1990)	Rehabilitation center	26 patients/8 weeks	Bandura scale of well-being/correlation between quality of life improvement and practice of limb strengthening exercises
Burkow *et al.* (2015)	At home – online coaching	10 patients/9 weeks	SGRQ score/enhancement of the scores of physical activity, decrease of symptom’s scores after rehabilitation
Güell *et al.* (2000)	Rehabilitation center	60 patients/24 weeks	CRQ score/improving dimensions = dyspnea, fatigue, emotional well-being, and autonomy after rehabilitation
Hansen *et al.* (2020)	Rehabilitation center versus at home	67 versus 67 patients/22 weeks	EQ-5D-5L and CCQ/milder anxiety and depression, non-inferiority between the two protocols
Nolan *et al.* (2019)	Rehabilitation center versus at home	154 versus 154 patients/8 weeks	CRQ score/superiority of the rehabilitation in a dedicated center. Improving dimensions = dyspnea, emotional well-being, and autonomy
Our study	At home	74 patients/16 weeks	SF-36 score/improving dimensions = physical capacity – role limitations due to emotional problems – general health

SGRQ = Saint Georges Respiratory Questionnaire; CRQ = COPD respiratory questionnaire; EQ-5D-5L = EuroQol 5 Dimension 5 Level; CCQ = Clinical COPD Questionnaire; SF-36 = Short form 36.

## Conclusions

According to experts, applying a RR protocol remains a cornerstone within the “Gold Standard” non-pharmacological treatment recommended for COPD. The most secure and codified way to perform it is to follow a personalized protocol in a dedicated center (Young, 1983; Zanotti *et al.*, 2012; Gloeckl *et al.*, 2013; Li *et al.*, 2013; He *et al.*, 2015; José and Dal Corso, 2016). These protocols are therefore applicable to a “vulnerable” population with advanced diseases. Physicians start usually by an assessment of the ventilatory, cardiac, and metabolic performances at rest and during effort. Then, the rehabilitation protocol would be “customized” for each patient defining the intensity and power levels requested.

However, we note a growing shift toward home rehabilitation protocols by practitioners, trying to adapt to precise kind of patients (Cameron-Tucker *et al.*, 2014; Marquis *et al.*, 2015; Vorrink *et al.*, 2016; Kwon *et al.*, 2018; Lahham *et al.*, 2018; Rassouli *et al.*, 2018). RR protocols at home are applicable to a wider and larger audience, providing necessary education and demonstration in order to convince patients. To guarantee safety, practitioners should make a careful selection of the targeted audience and avoid patients with heavy co-morbidities. The protocol is less strict, given the possibility of varying the intensity of exercise by patients themselves according to the subjective perception of dyspnea during exercise. Adherence to this protocol requires initial encouragement and a relationship of trust between physicians and patients. The application of a home-based RR is thought to maintain and improve self-confidence and quality of life of COPD patients compared to “classical” rehabilitation at a medical center.
